# Vehicle-assisted ligature decapitation – an unusual case report

**DOI:** 10.1007/s12024-023-00744-w

**Published:** 2023-12-19

**Authors:** Deniz Passos, Eduarda Duarte, Alexandra Andrade da Costa, Cátia Viana, Dina Almeida

**Affiliations:** National Institute of Legal Medicine and Forensic Sciences - North Branch, Jardim Carrilho Videira, 4050-167 Porto, Portugal

**Keywords:** Vehicle-assisted decapitation, Complex suicide, Forensic autopsy

## Abstract

Suicide by vehicle-assisted strangulation resulting in decapitation is a rare occurrence, characterized by a high kinetic energy mechanism that produces a clean-cut appearance in the decapitation area. Often resembling an incisive wound, this particular finding can mislead local authorities into investigating the case as a homicide. This case report describes an adult male who accelerated his vehicle after tying a nylon rope around his neck and securing it to a metallic structure on the wall. Furthermore, we conducted a brief review of cases published within the last ten years, summarizing the most prevalent findings associated with these incidents. By analyzing previously reported cases alongside our own, we aim to consolidate the prevailing patterns observed in vehicle-assisted strangulation cases. This underscores the paramount importance of thorough scene analysis by the medico-legal team and emphasizes the significance of subsequent necropsy findings in accurately discerning the manner of death.

## Introduction

Suicide by vehicle-assisted strangulation is an unusual and scarcely documented phenomenon, as evidenced by the limited number of published cases available to date. In such instances, the victim employs a ligature, securing it to a fixed point and subsequently placing it around the neck while inside a vehicle. Proceeding to accelerate the vehicle, in certain scenarios, can result in the complete decapitation of the victim. As this is a rare event and can produce a clean-cut appearance in the decapitation area, it can prompt local authorities to consider a homicidal manner of death and other different traumatic mechanism of lesions. We present a case report of a suicide by vehicle-assisted strangulation resulting in complete decapitation, involving a 49-year-old male victim. This report showcases the relevant scene examination procedures, morphological necropsy findings, and ancillary exams that helped the medico-legal experts instruct local authorities regarding the specific lesion mechanisms, assisting them in differentiating between a homicidal or a suicidal manner of death.

## Case report

**Fig. 1:**
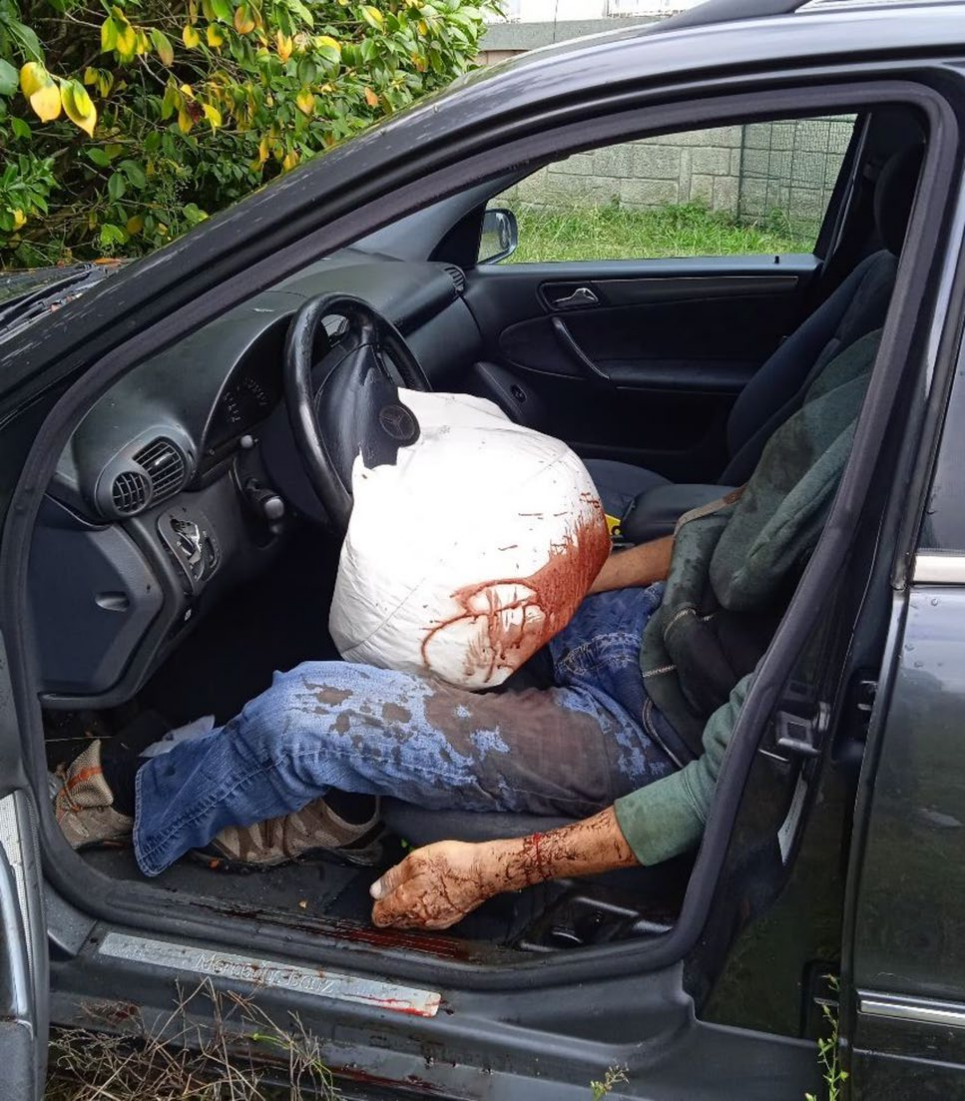
Body of victim inside vehicle

**Fig. 2:**
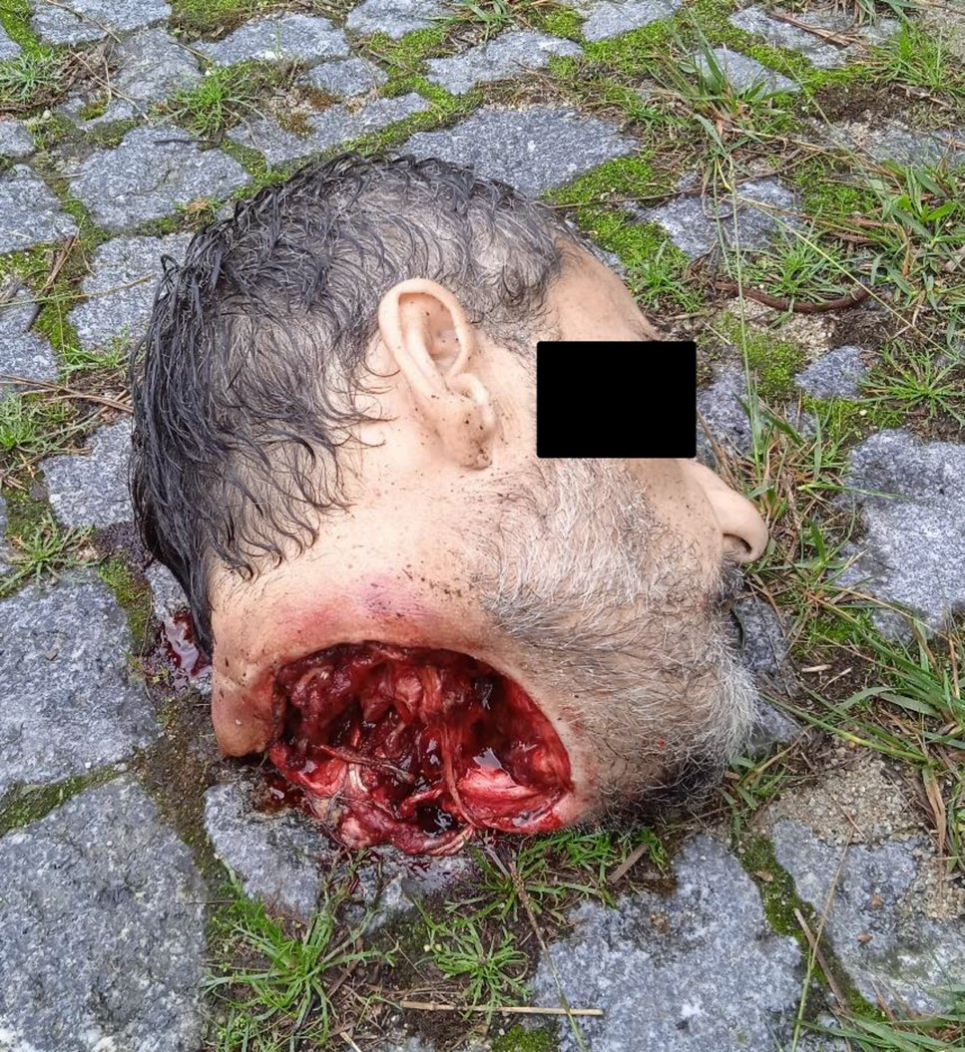
Victims head

**Fig. 3:**
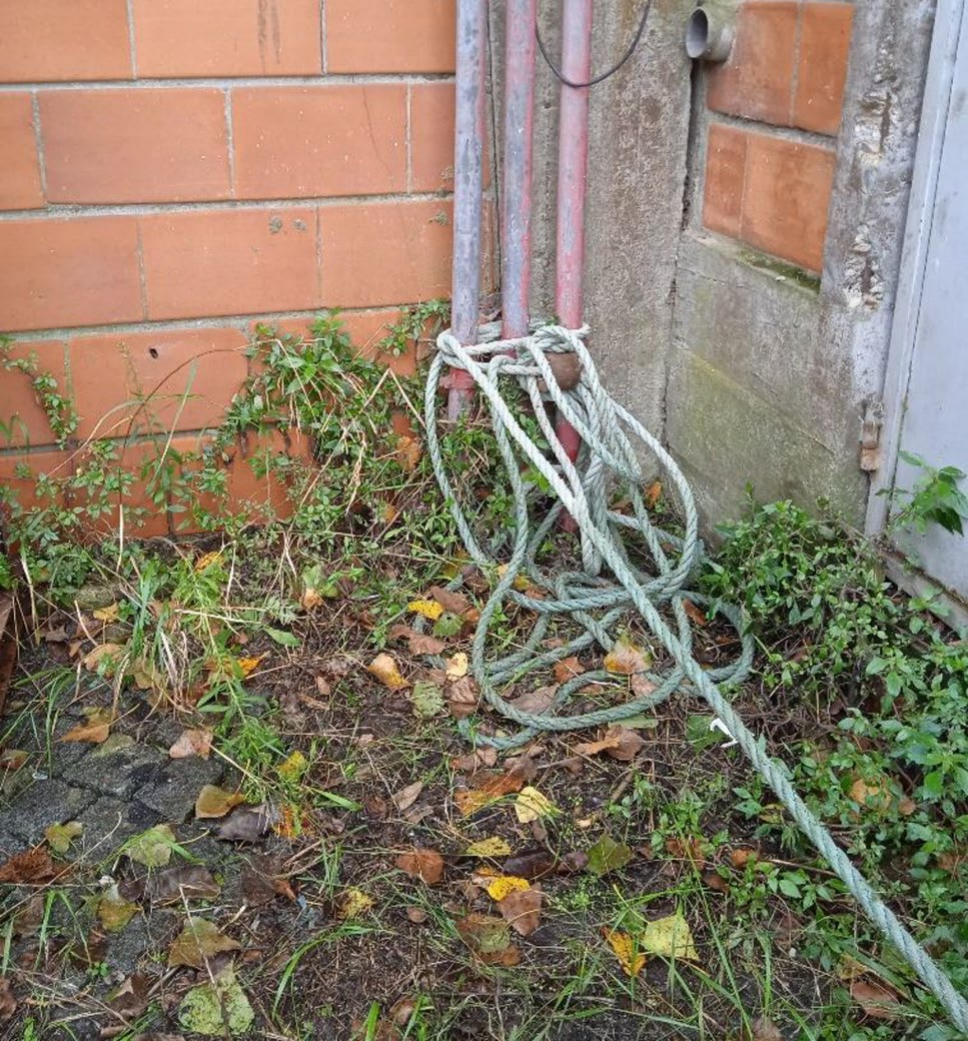
Nylon rope fixed to metallic structure

**Fig. 4:**
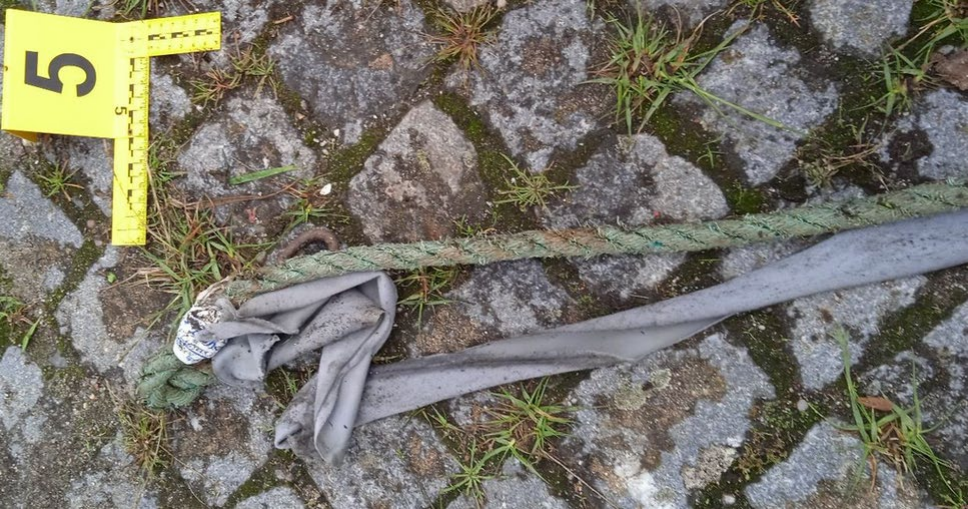
Nylon rope closed loop

A 49-year-old male, previously afflicted by recurring episodes of depression, experienced heightened symptoms attributed to financial challenges. There had been no documented instances of prior suicide attempts. His body was discovered within an abandoned industrial complex, situated inside a frontally crashed passenger vehicle. Airbags had been deployed, the key remained in the ignition, but the engine was inactive. The body, seated in the driver’s seat, was completely decapitated (see Fig. 1). The interior of the vehicle was soiled, predominantly on the left side, with the victim’s blood and soft tissues. Additionally, friction marks were identified on the upper part of the left back seat. A box cutter with the blade exposed was located on the passenger seat. Furthermore, the trunk was ajar, and various vehicle components, including the driver’s side headrest, parts of the trunk lamps, and other plastic elements, were scattered on the ground behind the vehicle. The victim’s head was discovered outside, lying on the ground approximately 10 m behind the vehicle (see Fig. 2). Upon a preliminary examination of the body, a clean-cut lesion was observed on the anterior neck skin. A recent incisive wound was also identified on the victim’s left wrist. On the opposite side of this industrial complex, on a slightly steep hill, a nylon rope was found attached to a metallic fixture on the wall (see Fig. 3). The rope, measuring approximately 20 m in length, featured a closed loop at its end, with remnants of soft tissues later confirmed to originate from the victim (see Fig. 4). Analysis conducted by local authorities on the tire marks revealed that the vehicle had travelled in a straight trajectory for about 42 m, from the location of the rope to the eventual crash site.

Inside the abandoned industrial building, the victim’s belongings were found, including a handwritten letter bearing the message “do not forgive me,” alongside multiple antidepressant tablets.

The initial assessment of the scene findings by the medico-legal team proved pivotal in the subsequent autopsy and in determining the manner of death, as local authorities had initially suspected foul play, considering this incident to be a potential homicide.

**Fig. 5:**
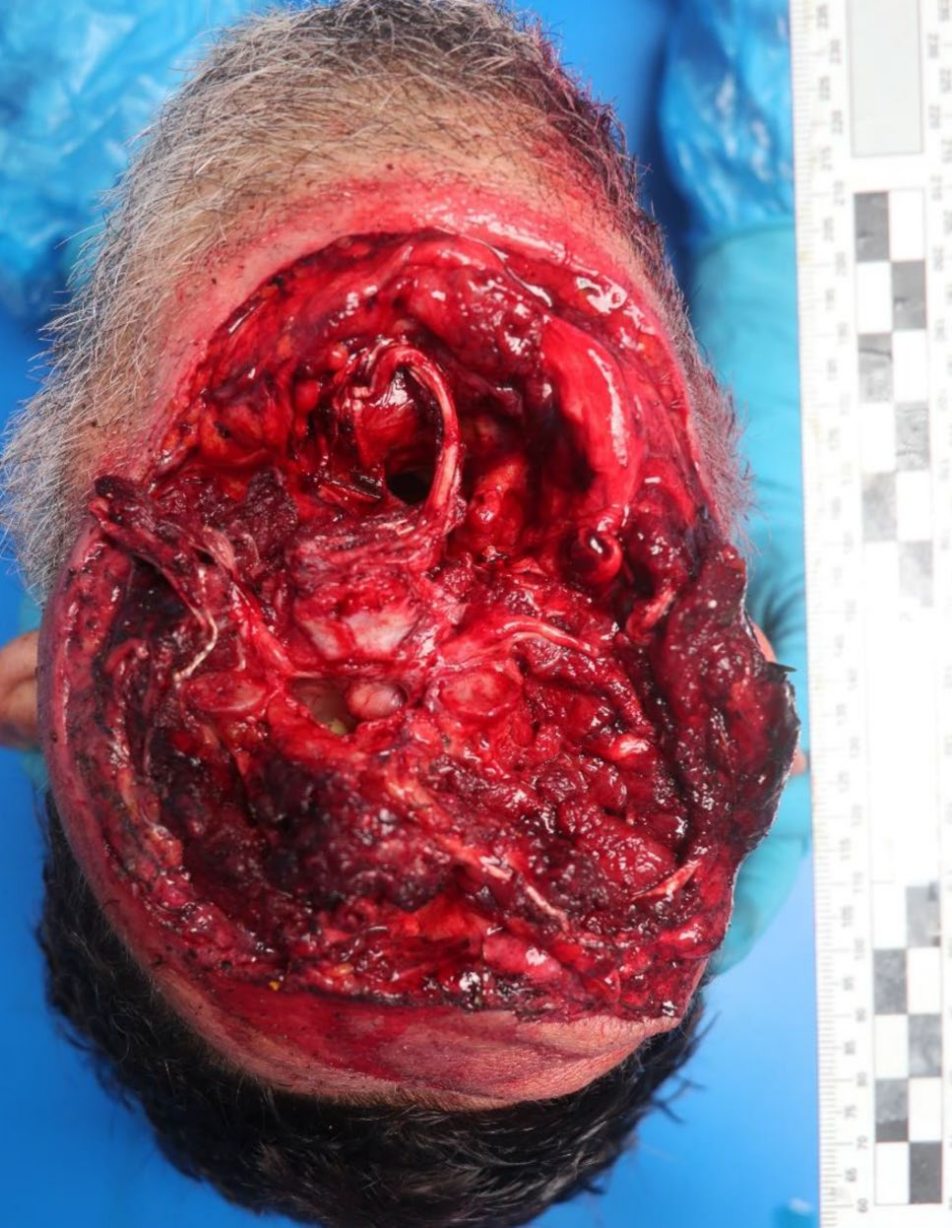
Inferior perspective of the decapitation injury

**Fig. 6:**
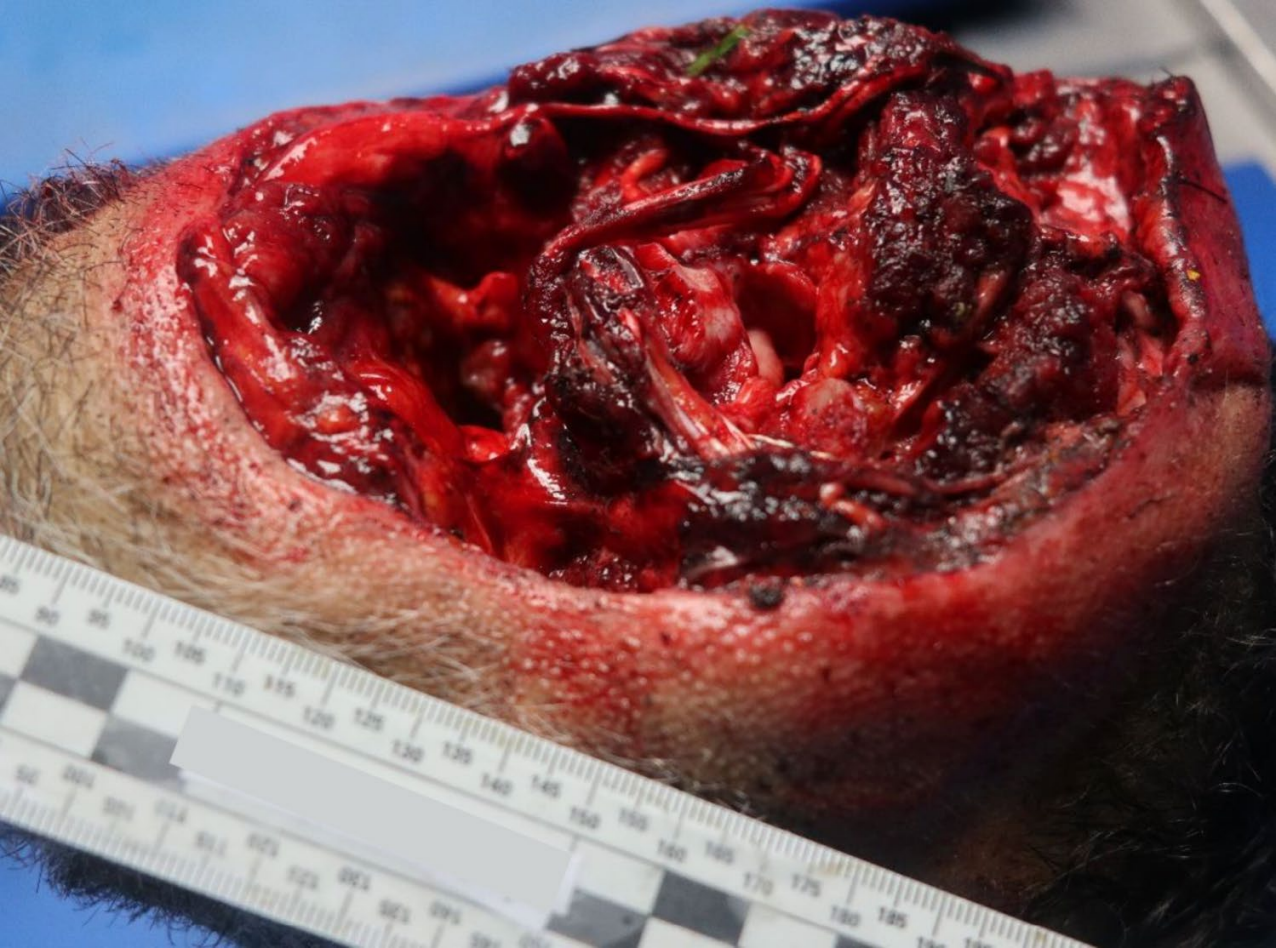
Decapitation injury at the right side of the head

**Fig. 7:**
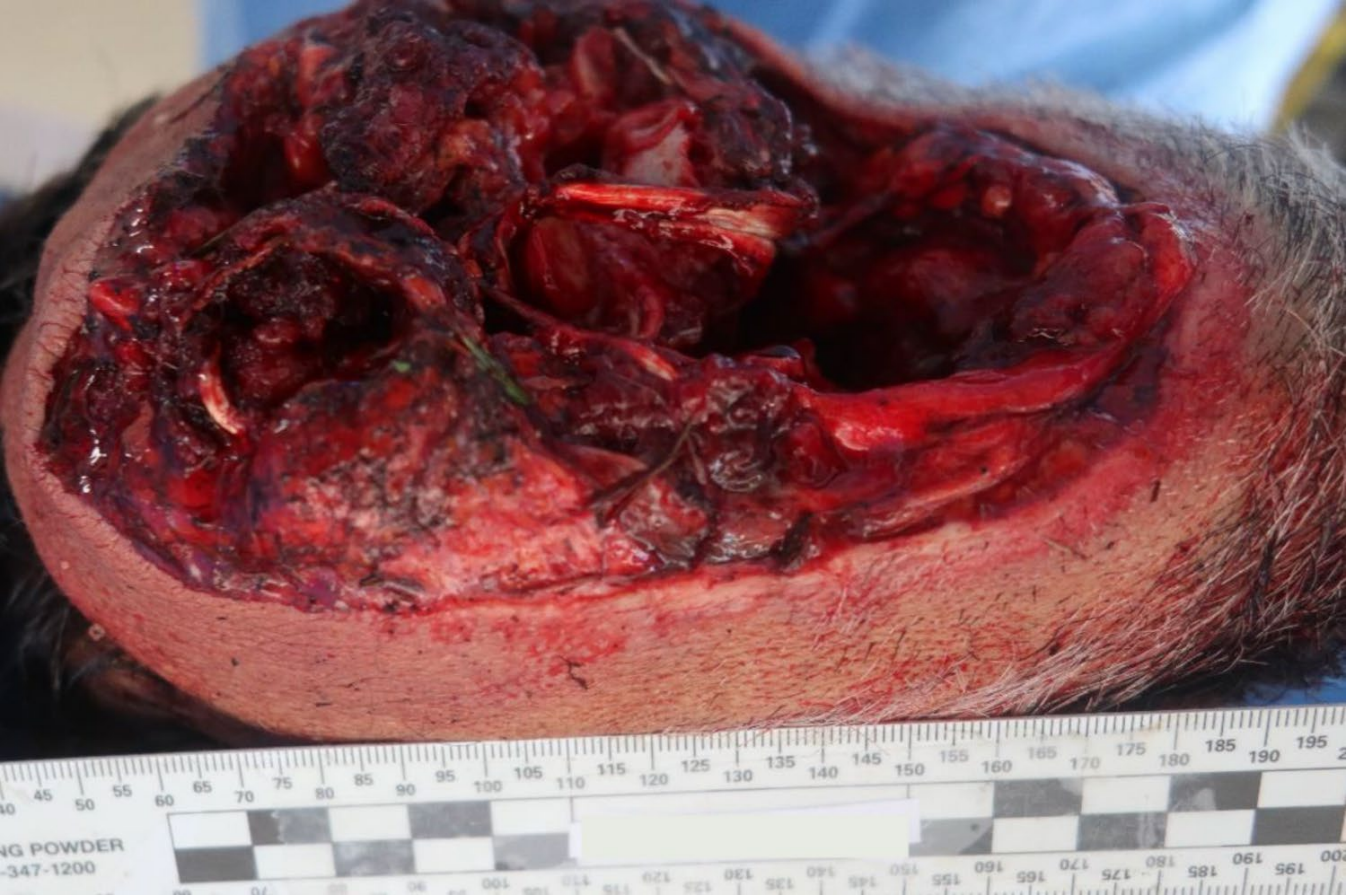
Decapitation injury at the right side of the head

**Fig. 8:**
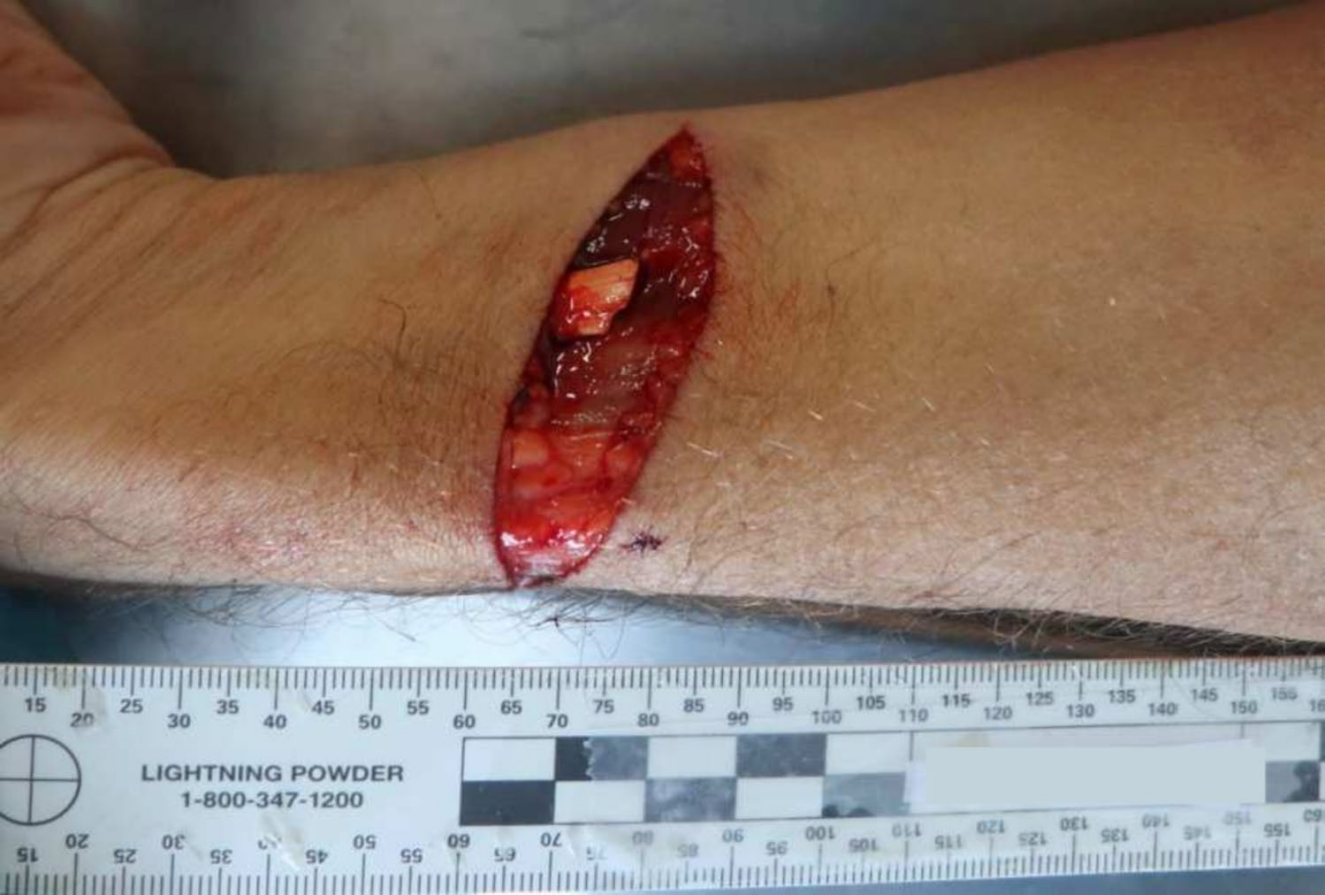
Upper perspective of cervical area

Autopsy was performed 2 days after death. The external examination of the victim revealed complete decapitation at the upper third of the cervical spine region. In this area, the skin exhibited blood infiltration, accompanied by a reddish abrasion measuring 2.5 cm in width on the right lateral surface, consistent with ligature marks. The lesion displayed clean-cut edges on the anterior and lateral surfaces, while irregular edges were noted on the posterior surface. There was also an associated laceration with hemorrhagic infiltration of all the inner soft tissues and structures, including a fracture of the thyroid cartilage and complete disarticulation at the C1/C2 level (refer to Figs. 5, 6 and 7). Furthermore, the hyoid bone and thyroid gland were absent from the body, presumed to have been lost at the scene (there was intense rain the day before the body was discovered). The head presented with multiple abrasive, brownish lesions, consistent with postmortem injuries possibly resulting from the impact and rolling of the head on the ground. An incisive lesion was also discovered on the antero-lateral surface of the left forearm, with hemorrhagic infiltration and section of muscular tissue, indicating an antemortem lesion inflicted by a blade, such as the box cutter present at the scene (see Fig. 8).

The toxicological analysis of cardiac blood indicated the presence of antidepressants within therapeutic levels, as well as the presence of paracetamol. Histological examination of the soft tissue found on the ligature confirmed it as human, presumably belonging to the victim.

In this case, the necropsy findings were consistent with the traumatic mechanisms observed, and the cause of death was attributed to the traumatic cervical injuries resulting from vehicle-assisted strangulation. Thus, the manner of death was determined to be suicide.

## Discussion

In ligature strangulation, death usually results from a combination of airway occlusion, neck veins and artery occlusion, and nerve effects [6]. In instances of vehicle-assisted ligature decapitation, a ligature is placed around the victim’s neck and secured to a point outside the vehicle. While inside the vehicle, the high kinetic energy of the ligature generated by the acceleration of the vehicle on a downhill slope, and the nature of the ligature, leads to rapid tearing of cervical tissues and a subsequent clean-cut appearance of cervical lesions. In cases like this one, complete decapitation can occur.

There are limited published cases available to date. In order to compile the most common findings in these rare cases of decapitation, a concise literature review was conducted. The search was performed on PubMed, focusing on original case reports and publications from the last 10 years, encompassing articles since 2012. The following research keywords were applied: “ligature assisted decapitation.” From this analysis, 5 original case reports of vehicle-assisted strangulation were identified, among which only 4 involved complete decapitation of the victim. The findings from these cases are summarized below [1–4].

All the cases identified had a suicidal manner of death and involved adult males. Similar to our case report, the victims was usually found inside the vehicle, in the driver’s seat, with the head remaining inside the vehicle. Notably, our case differed in that the head was discovered outside the vehicle, likely due to the fact that the trunk was open. In most instances, including our case, the vehicles sustained some form of damage, such as bloodstains, broken headrests, or shattered windows. In every case, a rope was discovered, fastened to a fixture some distance away from the vehicle. The decapitation area was consistently described “severed straight,” “sharp cut,” or “clear-cut.” These findings can pose challenges for local authorities, as they often mimic incisive wounds, potentially raising suspicions of a homicidal manner of death (see Table 1).

**Table 1:** Case reports since 2012 findings

**Year of publication**	**Article title**	**Victim characterization**	**Relevant findings**
2012	“Motor vehicle-assisted ligature strangulation causing complete decapitation: an autopsy report”	63-year-old male	Body found decapitated on the driver’s seat in a van on a downhill road, with the head stuck in between the steering wheel, the dashboard, and the driver-side door Skin at the decapitation site was “clear-cut,” with “multiple imprints of the nylon rope.” Nylon rope found tied to a gate, 60 m from the vehicle
2012	“Vehicle-assisted decapitation a case report”	40-year-old male	Body found decapitated on the driver’s seat of a crashed car, with the head on the back seat Skin at the decapitation site was “severed straight” and presented a “strangulation line” Rope (11 mm in diameter) found tied to a tree, 90 m from the vehicle
2012	“Different mechanisms of decapitation: three classic and one unique case history”	Age not specified, male	Body found decapitated on driver’ seat of a car, with the head between the front seats Skin at the decapitation site was a “relatively sharp cut wound” on the front and “torn off” on the back. Soft tissue was irregularly cut Nylon rope (7 mm in diameter) found tied to a light post, 55 m from the vehicle. Soft tissue on ligature
2019	“Vehicle-assisted ligature decapitation: a case report and a review of the literature”	43-year-old male	Body found decapitated on the driver’s seat of a car, head on the front passenger seat Skin at the decapitation site was “clear-cut” and “slightly abraded” on the posterior surface Steel rope (5 mm in diameter) found tied to a light post, 100 m from the vehicle

All of these findings align with those in our case report, thereby reinforcing the evidence that a clean-cut appearance is a prevalent characteristic in ligature-assisted decapitations. This serves to mitigate the confusion sometimes associated with this specific finding, reducing the risk of misdiagnosis, particularly in differentiating it from incisive wounds.

One of the primary objectives of a medico-legal autopsy is to ascertain both the cause and manner of death, with the latter often reliant on the analysis of circumstantial evidence [7]. Some cases of suicide can be unusual and even extraordinarily bizarre. In the present case, a self-inflicted non-fatal wound was identified on the left wrist, distinct from “tentative incisions.” This is a common injury observed in suicide cases [6].

In cases of ligature strangulation, the ligature mark typically encircles the neck horizontally. However, in this extreme case, the presence of decapitation alongside tissue destruction made evaluating the ligature mark particularly challenging. Signs of cranial congestion were not present in this case, a circumstance that aligns with the swift and forceful nature of the incident.

The forensic on-site examination of a body plays a pivotal role, particularly in situations where the cause of death is presumed to be suspicious. This examination is crucial as it facilitates the accurate collection and identification of evidence, providing vital information for the subsequent stages of investigation, It forms an integral part of the forensic investigation process and significantly contributes to the proper execution of the medico-legal autopsy [5]. The particular finding of a clean-cut appearance in neck lesions initially led local authorities to suspect a murder had occurred, as these findings resembled incisive wounds. Given the limited circumstantial information available, the thorough analysis of the death scene by the forensic pathologists provided substantial evidence supporting the likelihood of a suicide, assisting in enlightening both local authorities and the court as to the manner of death.

## Key points

1. Suicide by vehicle-assisted strangulation is unusual and scarcely documented.
2. Local authorities may misdiagnose these cases as a homicidal manner of death.
3. Forensic on-site examination of the body plays a pivotal role.
4. Typical lesions include a clean-cut appearance in the decapitation area.
5. Thorough analysis of the death scene and necropsy assist in enlightening to a suicidal manner of death.
